# Realization of pitch-rotational torque wrench in two-beam optical tweezers

**Published:** 2021-11-26

**Authors:** Muruga Lokesh, Rahul Vaippully, Vidya P Bhallamudi, Anil Prabhakar, Basudev Roy

**Affiliations:** 1 Department of Physics, Indian Institute of Technology Madras, Chennai, 600036, India; 2 Department of Electrical Engineering, Indian Institute of Technology Madras, Chennai, 600036, India

**Keywords:** optical tweezers, pitch rotation, pitch rotational torque wrench

## Abstract

3D Pitch (out-of-plane) rotational motion has been generated in spherical particles by maneuvering the laser spots of holographic optical tweezers. However, since the spherical particles, which are required to minimise drag are perfectly isotropic, a controllable torque cannot be applied with it. It remains free to spin about any axis even after moving the tweezers beams. It is here that we trap birefringent particles of about 3 *μm* diameter in two tweezers beams and then change the depth of one of the beam foci controllably to generate a pitch rotational torque-wrench and avoid the free spinning of the particle. We also detect the rotation with newly developed pitch motion detection technique and apply controlled torques on the particle.

## 1 Introduction

Optical tweezers provides a simple, robust and highly efficient platform to manipulate microscopic particles [1–3] both in translational and rotational degrees of freedom. Translational manipulation of micron sized particles and applying piconewton forces is a well desired feature in a wide variety of fields starting from biology [4–6] to physics [7–10] to even chemistry [11–13]. In addition, trapped birefringent particles are rotated utilizing the polarization [14–16] of the trapping laser. A single biological specimen like a protein or a DNA [17] can be attached to a trapped particle to disclose the dynamics and estimate torques [18] or forces [19].

A trapped particle has three degrees of rotational freedom, two of which corresponds to out of the plane rotations [20] (pitch, roll) and one in-plane rotation [21] (yaw). Many techniques have been illustrated to rotate trapped particles using orbital angular momentum beams [22–24], holographic optical tweezers [25–27] and controlling ellipticity of the beams[14]. These techniques explore yaw rotational motion explicitly. On the other hand, controlled generation of pitch motion has also been realised in previous work [28, 29] where, video microscopy was employed to affirm the generated motion of asymmetric, non-birefringent particles [30, 31]. Surfaces have been moved to turn particles optically trapped but adherently residing on them [32]. Entire cells have been rotated in the pitch sense which is quite useful for tomography [27]. Moreover, the pitch motion shows different dynamics in proximity to surfaces than yaw motion.

When rotated in the pitch sense, the non-birefringent particles can spin uncontrollably due to the lack of orientational confinement of the particle. The orientational confinement is very important in probing problems like quantum sensing using Nitrogen-Vacancy (NV) centers in diamonds. Further, if one has to apply a shear force on a membrane using a particle, this pitch mode is an alternative to translating the particle along the surface, which can encounter less Stokes drag. The rotational case merely amounts to a fraction of one complete rotation of the particle while the translational case amounts to the entire center of mass moving by a larger extent. This assumes significance when faster dynamics is being studied.

Here, we apply controlled pitch torques and also employ a high resolution pitch-detection system [33] to calibrate it in spherical birefringent particles. This scheme of two-beam confinement applies constant pitch torques on a trapped birefringent particle of about 500 pN-nm. The detection system measures the asymmetry in the scattered pattern under cross-polarizers. This technique is prominent as it provides control over an additional rotational degree of freedom and allows us to explore complex dynamics.

## 2 Theory

**Figure 1:**
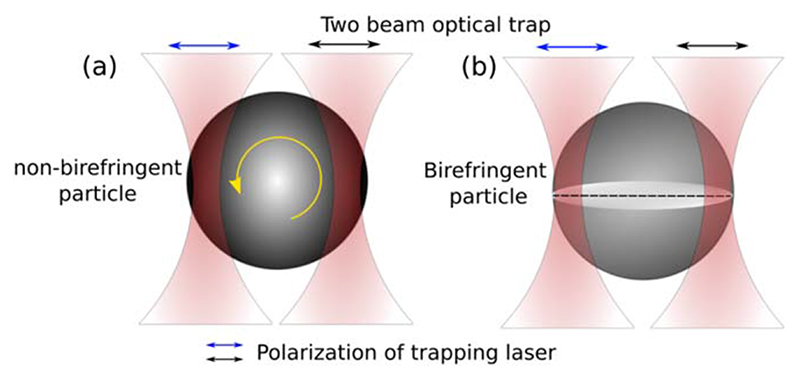
Schematics of two tightly focused lasers beams with same polarization (shown in blue and black arrows) simultaneously trapping (a) a spherical non-birefringent particle showing translational confinement but freedom to rotate(yellow), (b) a spherical birefringent particle confined both in translational and rotational degrees.

A non-birefringent spherical particle trapped with two traps as depicted in the figure 1 (a) is not rotationally confined. Birefringent particles tend to orient to the direction of polarization of trapping laser breaking the rotational symmetry.

The electric field **E** incident on the birefringent particle experiences different refractive indices *n_o_* and *n_e_* along ordinary and extraordinary axis. The electric field **E** of the trapping laser induces a polarization **P** on the particle and interacts with the induced polarization to produce a torque (*τ*) given by [34].

(1) $$\tau =\int^{\text{​}}d^{3}x(P\times E)$$

A restoring torque per unit area (*τ*) is produced due to the change in angular momentum of the elliptically polarized light passing through the birefringent particle of diameter d_1_ given by equation (2), [14]

(2) $$\begin{array}{ll} \tau = & -\frac{\epsilon }{2\omega_{1}}E^{2}\sin\left(kd_{1}\left(n_{0}-n_{e}\right)\right)\cos2\phi \sin2\theta \\ & +\frac{\epsilon }{2\omega_{1}}E^{2}\left\{1-\cos\left(kd_{1}\left(n_{o}-n_{e}\right)\right)\right\}\sin2\phi \hat{n} \end{array}\text{​}$$

where, *ϕ* is the degree of ellipticity of light, *k* is free space wavenumber, *ϵ* is the permittivity, θ is the angle between the birefringent axis and trap polarization, and ω_1_ is the angular frequency of electric field. The expression is derived in detail in the reference [14].

**Figure 2:**
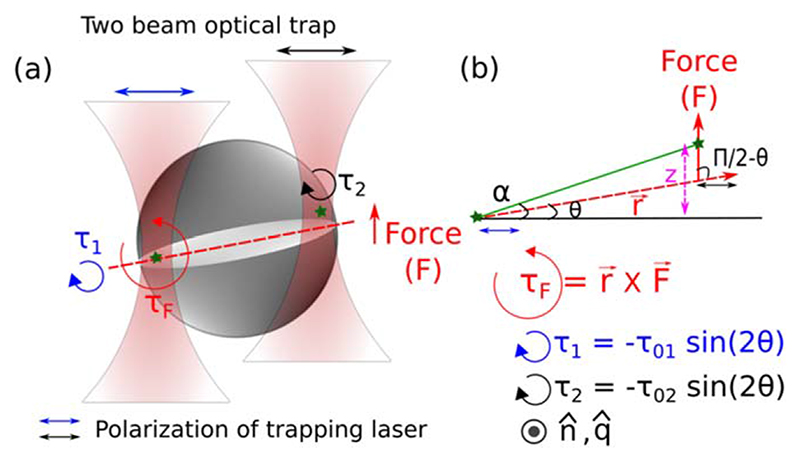
(a) Schematics of a spherical birefringent particle in stable configuration depicting torques (*τ*_1_, *τ*_2_ and *τ*_F_) and force F when trapped by two tightly focused lasers beams with same polarization (shown in blue and black arrows). The torques τ_1_ and *τ*_2_ arise due to the misalignment of polarization (P) with the electric field (E). Whereas, the torque (*τ*_F_) is generated by the optical force (F) applied by one trap while the other trap act as a pivot. (b) Vector representation of torques and force acting on the birefringent particle.

As *ϕ* → 0, the torque for linearly polarized light becomes

(3) $$\begin{array}{ll} \tau & =-\frac{\epsilon }{2\omega }E^{2}\sin\left(kd_{1}\left(n_{0}-n_{e}\right)\right)\sin2\theta \hat{n}\\ & =-\tau_{0}\sin2\theta \hat{n} \end{array}$$

where, *τ*_0_ is the maximum torque on the particle and $\hat{n}$ is the direction of torque perpendicular to both **E** and **P**. The particle experiences torques *τ*_1_ and *τ*_2_ given by equation (4), (5) due to the interaction of induced dipoles **P**_1_ and **P**_2_ with corresponding electric fields **E**_1_ and **E**_2_ at each trap respectively.

(4) $$\tau_{1}=\int^{\text{​}}d^{3}x\left(P_{1}\times E_{1}\right)=-\tau_{01}\sin2\theta \hat{q}$$

(5) $$\tau_{2}=\int^{\text{​}}d^{3}x\left(P_{2}\times E_{2}\right)=-\tau_{02}\sin2\theta \hat{q}$$

where, *τ*_01_, *τ*_02_ are maximum torques that can be applied, $\hat{q}$ is the unit vector perpendicular to **P**_1,2_ and **E**_1,2_. Also, sin2*θ* dependence of the torque indicate that the particle has a two-fold symmetry. In addition, particle also experiences a torque (*τ*_F_) given by equation (6) due to the optical force (**F**) exerted by one trap arising from the intensity gradient while the other trap acts as a pivot as depicted in figure 2(b).

(6) $$\begin{array}{ll} T_{F} & =r\times F\\ & =|r||F|\sin\left(\frac{\pi }{2}-\theta \right)(-\hat{q})\\ & =r|F|\cos(\theta )(-\hat{q}) \end{array}$$

where, **r** is the vector joining two focal spots and $\hat{q}$ is unit vector perpendicular to **r** and gradient force **F** given by equation (7).

(7) $$\begin{array}{ll} F & =-\nabla \left(P_{2}\cdot E_{2}\right);\left[P_{2}=\epsilon_{0}\chi E_{2}\right]\\ & =-\nabla \left(\epsilon_{0}\chi E_{2}\cdot E_{2}\cos(\theta )\right)\\ & =-\epsilon_{0}\nabla \left(\epsilon_{r}-1\right)E_{2}^{2}\cos(\theta ) \end{array}$$

Note that the direction of *τ*_1_ and *τ*_2_, and that ofτ_F_ are in opposite directions and the total torque (*τ*_T_) is equal to sum ofall torques acting on the particle.

(8) $$\begin{array}{ll} \tau_{T} & =\tau_{1}+\tau_{2}+\tau_{F}\\ & =-\left[\left(\tau_{01}+\tau_{02}\right)\sin2\theta +rF\cos(\theta )\right]\hat{q}\\ & =-\left[2\left(\tau_{01}+\tau_{02}\right)\sin\theta \cos\theta -r\epsilon_{0}\frac{\partial }{\partial z}\left[\left(\epsilon_{r}-1\right)E_{2}^{2}\right]\cos^{2}(\theta )\right]\hat{q}\\ & =-\cos\theta [A\sin\theta -B\cos\theta ]\hat{q}\\ & =-\sqrt{A^{2}+B^{2}}\cos\theta \sin(\theta -\beta )\hat{q}\\ & =-\left(\frac{\sqrt{A^{2}+B^{2}}}{2}\right)(\sin(2(\theta -\beta /2))-\sin(\beta ))\hat{q} \end{array}$$

where,A = 2(*τ*_01_ + *τ*_02_),$\text{B}=r\epsilon_{0}\frac{\partial }{\partial z}\left[\left(\epsilon_{r}-1\right)E_{2}^{2}\right]=\kappa_{z}\text{Z},\kappa_{z}$ is trap stiffness in *z* direction and the particle tries to align to minimize torque. Here, tan $\beta =\text{B}/\text{A}=\frac{z}{z_{0}}$ and $z_{0}=\frac{2\left(\tau_{01}+\tau_{02}\right)}{\kappa_{z}}$. Then in the limit that the (*θ* — *β*) is small, we get,

(9) $$\tau_{T}=-\sqrt{A^{2}+B^{2}}\cos(\beta )(\theta -\beta )\hat{q}$$

Thus there is a restoring torque trying to align the particle towards the angle β. It also implies that the magnitude of the torque is maximum when *β* = 0°, while it is negligible when the *β* = 90°.

## 3 Experimental procedure

**Figure 3:**
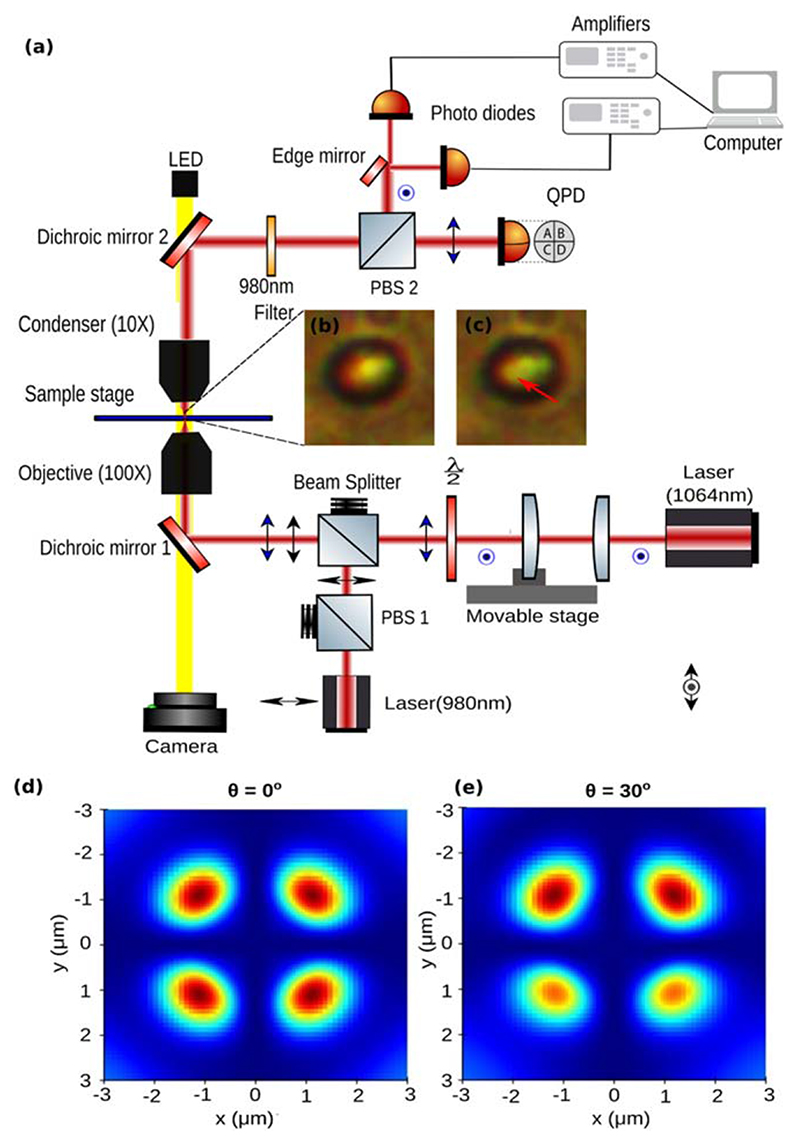
(a) Schematic diagram of the setup with the spherical particle simultaneously trapped by two tightly focused lasers (b) Image of the birefringent particle trapped by two beams in the first configuration, (c) which then turns to the other configuration. (d) The simulated scatter pattern for a birefringent particle symmetrically oriented with the crossed polarizers. (e) The simulated scatter pattern with the pitch angle turning by 30°. Details of simulation in [35].

We show the experimental setup to generate and detect pitch motion as figure 3. We use the OTKB/M Optical Tweezers kit from Thorlabs, U.S.A, to do the experiment. Birefringent particles suspended in 20 μl distilled water was placed between a glass slide (Bluestar, 75mm× 25mm× 1.1mm)and a coverslip (Bluestar, number 1 size, english glass) to form the sample chamber. An oil immersion 100×, 1.3NA objective from Olympus with inverted microscope configuration is used trap the birefringent particles as shown in figure 3. The scattered beam is then collected using a condenser lens objective (E plan 10×, 0.25 air-immersion) (Nikon). A dichroic mirror and polarizing beam splitter(PBS) directs most of the scattered light into a quadrant photodiode (QPD) to detect translational motion of the trapped particle. Also, a white light source illuminates the sample chamber from the top coupled with dichroic mirrors and the light is directed to a CMOS camera as shown in figure 3.

Two diode lasers of wavelengths 1064nm and 980 nm were used in the experiment and are focused onto the sample plane via two different paths combined using a beam splitter. A set of lenses are mounted on a movable stage to change the position of the 1064nm trap. A polarizing beam splitter and half-wave plate were used in beam paths of 980 nm and 1064nm lasers respectively to match the polarizations at the sample plane. A 980 nm filter was used after the Dichroic mirror 2 to isolate the 980 nm light from the 1064nm light.

The 980 nm beam is held fixed and acts as the reference for turning the particle. The 1064 nm beam focus is moved which in-turn moves the particle. The detection of the angle requires the scatter pattern under crossed polarizers at only one wavelength. Using both 980 nm and 1064 nm for detection can cause complications in the detection, which may give imperfect results. It is not known what the exact effects will be if both are used simultaneously but are expected to cause complications. Hence the 980 nm light is filtered and used.

The pitch angle determination unit works by finding the asymmetry in the scatter pattern of a spherical birefringent particle under crossed polarizers. Finding the asymmetry requires the usage of a quadrant photodiode to find the difference in the intensities between the left half of the beam and the right half of the beam. Thus, we use the edge mirror and the two photodiodes.

The images in figure 3(b) and (c) makes the particle look different from a sphere because the imaging has been performed under crossed polarizers, so that the scatter pattern hardly shows the exact outline of the particle. The background also does not look perfectly dark. This is because we have used sheet polarizers and has imperfections. Moreover, the differences in the scatter pattern are small and hence the scattered tweezers light is itself used to ascertain the asymmetry. The typical scatter pattern with the birefringence axis symmetrically oriented and turned by 30 degrees is shown in figure 3(d) and (e). These patterns look different from the images because the visible light requires polarizers for those wavelengths which are the inefficient sheet polarizers in this case.

Birefringent particles used in the experiment were synthesized using RM257 (Merck) nematic liquid crystal precursor powder. The preparation protocol requires 99% pure ethanol (50 ml) and de-ionised water (150 ml) in 1:3 ratio which were heated to 55°Cand 75°Crespectively in separate beakers. Temperatures of ethanol and water were monitored and when, ethanol reaches 55° C, about 80 mg of RM257 powder was added. The solution is stirred with a small magnetic stirrer in the beaker allowing the powder to dissolve uniformly. Subsequently, this RM257-ethanol solution was added to de-ionised water in dropwise fashion. A milky white solution was formed in the beaker which then is closed using a perforated aluminium foil. Ethanol was evaporated from these perforations leaving the overall solution at 150 ml. Later, when the solution cools down to room temperature we can store the solution and use it for experiments. The particles sizes synthesized using this method varies from 2 *μ*m to 3 *μ*m.

The 980 nmlaser has a Polarizing Beam Splitter (PBS1) in front of the laser itself. The other polarizer (PBS2) is after the 980 nmfilter. These two ensure that when the scattered light passes through the output polarizer, it provides angular information.

The simulated scattered patterns a birefringent particle rotated in the pitch sense has been shown in figure 3(d) and (e). The methodology of the simulation has been shown in [35].

## 4 Results and discussions

Wetrap a birefringent particle simultaneously with two optical traps and move one of the traps which makes the particle move in pitch sense as depicted in figure 2. The out-of-plane movement of the spherical particle is hard to observe in video microscopy technique. However, the pitch motion of a birefringent spherical particle can be detected as the amount of asymmetry in scattered light collected with cross-polarizers [35]. The setup can be utilized to apply controlled torques on spherical particles.

When a birefringent particle is trapped between cross polarizers, the light scattered forms a symmetric four lobe pattern. When the particle turns in the pitch sense, an asymmetry appears by making two of the lobes glow brighter than the other two without effecting the total intensity. The amount of asymmetry is measured by taking the difference of intensities between two halves which is correlated to the pitch angle. Hence the pitch angle can be calculated based on the intensity difference of two halves of scattered light [33].

The exact value of the rotation angle canbe found by multiplying a calibration factor (*β*) derived from power spectral density [36] of the particle in the presence of the Brownian motion with the rotation signal obtained from the photodiode in volts.

**Figure 4:**
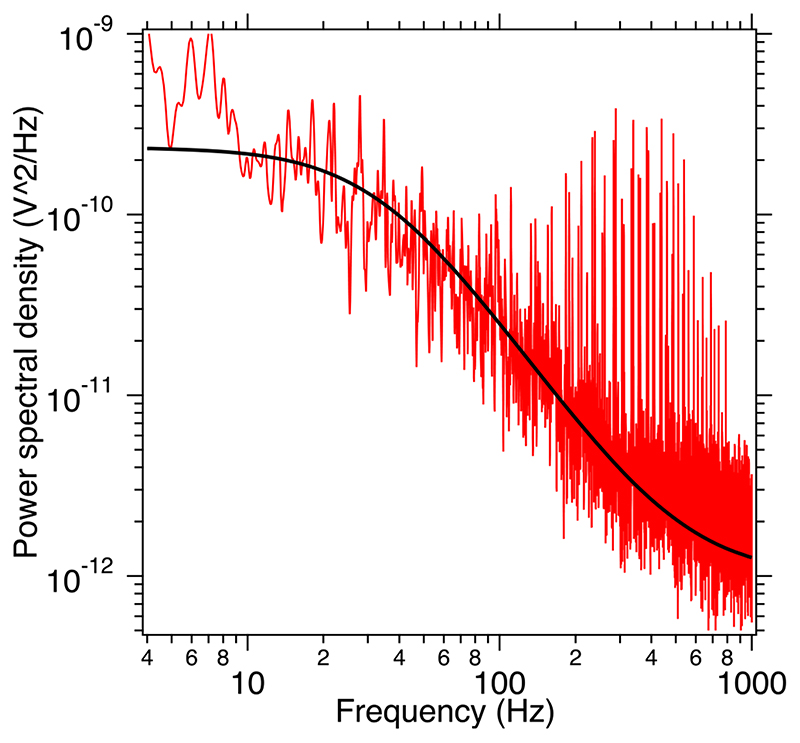
Pitch Power Spectral Density of the particle trapped in 2 beams fit to a Lorentzian (black) of the form in equation (10). The value of A = (2.6 ± 0.6) × 10^-7^ V^2^ Hz and B = 1134 ± 386 Hz^2^.

Pitch power spectral density (PSD) of a trapped particle follows a Lorentzian and is fit to equation (10) with the calibration factor (*β*) is given by equation (11) [36].

(10) $$\text{PSD}=\frac{A}{f^{2}+B}$$

(11) $$\beta =\frac{1}{2\pi }\sqrt{\frac{k_{B}T}{\gamma A}}$$

where, A and B are fitting parameters, *f* is frequency and *γ* is drag coefficient. The calibration factor *β* multiplied by the signal acquired from the photodiodes give us the pitch angle (*θ*) as a function of time and is shown in figure 4(a).

This controlled pitch motion is generated by changing the depth of one of the two traps using a movable lens, as shown in figure 3. The focus of the second trap is moved vertically in and then out such that first it moves clockwise and then counterclockwise in the pitch sense. Pitch angular frequency $\left(\omega =\frac{d\theta }{dt}\right)$ is calculated numerically from the pitch time series as follows :

(12) $$\omega =\frac{\theta (t+\delta t)-\theta (t-\delta t)}{2\delta t}$$

Pitch angle (*θ*) of the spherical particle is changing with a constant slope on the angle versus time graph, which implies a constant torque is being applied on the particle. Also, the torque (*τ*) applied on the particle can be calculated from the pitch angular velocity (*ω*) and viscous drag of the medium (*γ*) given by equation (13). Here, it may be noted that this system is an overdamped system where the inertial term is about 5 orders of magnitude larger than the damping term. The torque felt by the system is mainly computed from the drag.

(13) $$\tau =\gamma \frac{d\theta }{dt}=\gamma \omega$$

**Figure 5:**
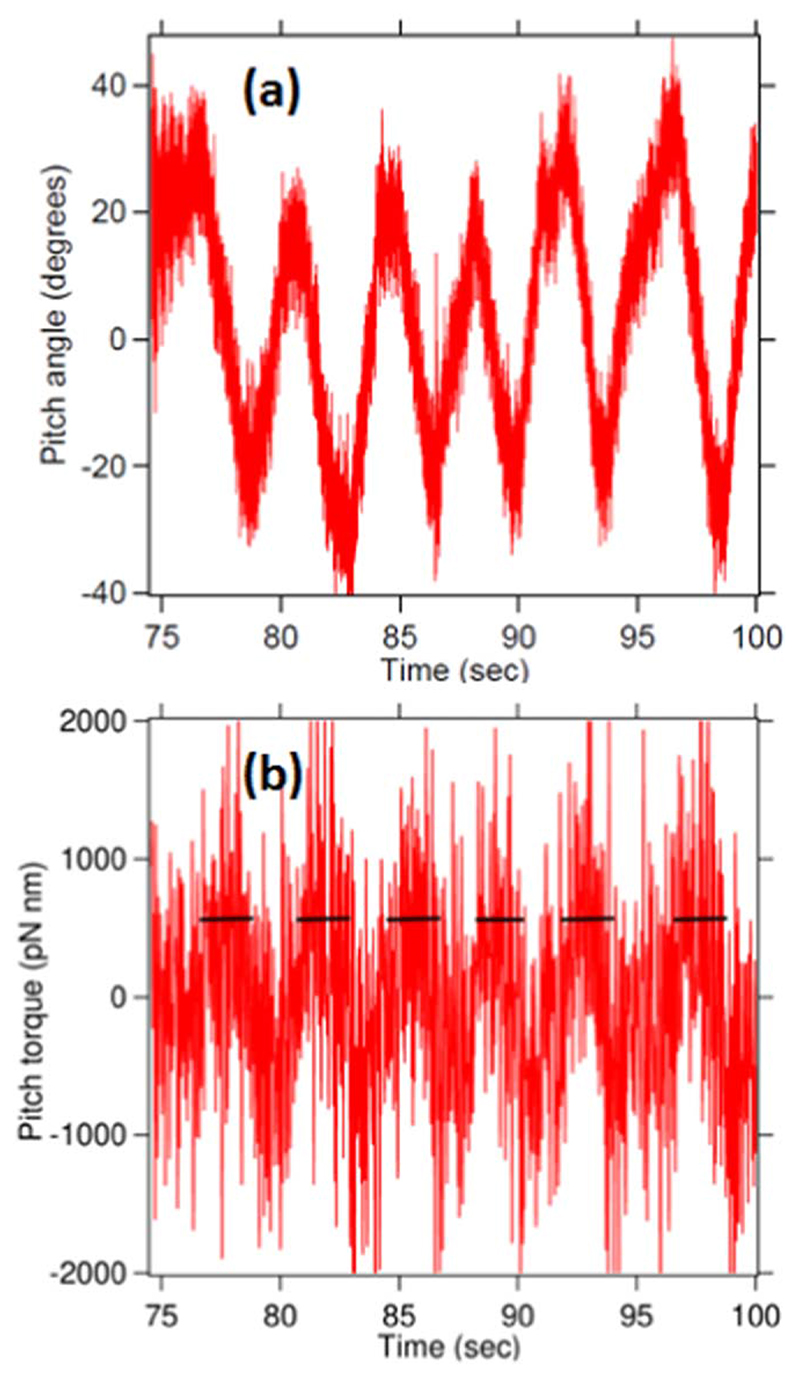
(a) This figure indicates the change in the pitch angle by moving the focus of one of the trapping beams. (b) The corresponding pitch torque applied on the trapped particle.

**Figure 6:**
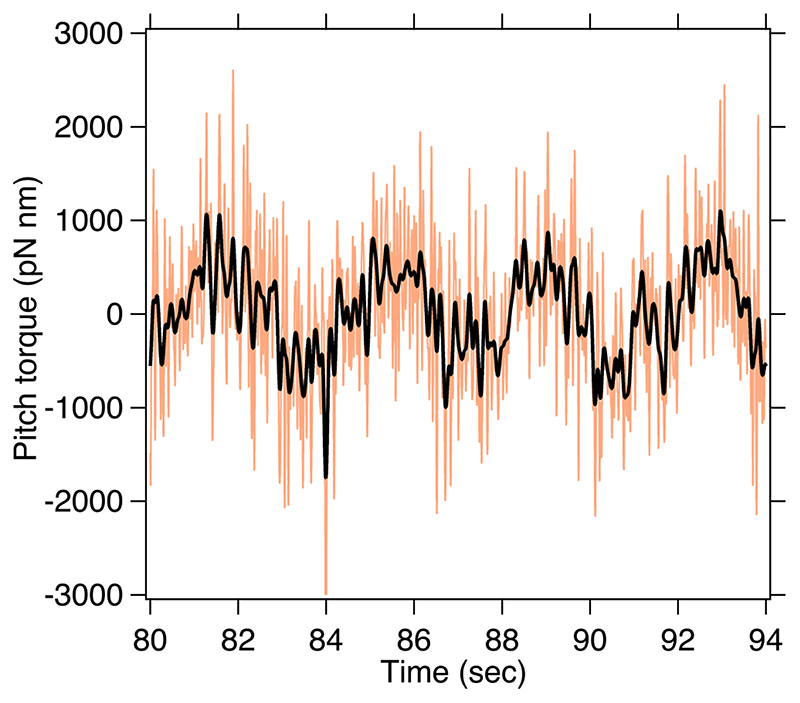
The figure is the zoomed in version of the figure 5(b) depicting the regions of constant pitch torque applied on the trapped particle. The smoothened data indicating constant regions of torque are also shown. The smoothened curve is a square wave function with imperfections due to manual motion of stage.

The plot shown in figure 5(b) applies constant torque which can be observed in the small regions highlighted with black lines. The constant torque was shown by zooming in as shown in figure 6. Hence, the system is acting as a torque wrench applying constant torques on the spherical birefringent particle. We also show a video of the execution of the pitch motion by using 2 beams in the supplementary video available at stacks.iop.org/JPCO/5/115016/mmedia S1.

This technique only works if the size of the particle is larger than two times the mode waist of the optical trapping beams. In our case, since the approximate wavelength is 1 μm, the particle diameter has to be at least 2 μm.

## 5 Conclusions

Thus, to conclude, we have successfully designed a torque-wrench to apply a constant torque of 500 pN-nm on a 3 *μm* spherical birefringent particle using two laser beams. We were also able to rotate the particle by 40° angle. The system can be used where controlled pitch torques are needed. It yields different dynamics from the yaw rotation particularly in proximity to surfaces. Hence the pitch torque can also have major applications when applying torques on soft surfaces like cell membranes and single proteins or DNA molecules attached to surfaces. This can be envisaged as a different mode of applying controlled stresses on molecules or surfaces. Even in the NV centers of diamonds, which have 4 precise orientations on the diamond lattice, the control over the pitch and yaw simultaneously may work wonders to the sensing capabilities of the particle.

## Supplementary Material

S1

## Data Availability

The data that support the findings of this study are available upon reasonable request from the authors.ORCID iDs
